# Infrasound Array Dataset of the 2021 Eruptive Paroxysms of Etna Volcano

**DOI:** 10.1038/s41597-026-06638-0

**Published:** 2026-01-24

**Authors:** Luciano Zuccarello, Duccio Gheri, Silvio De Angelis, Araceli Garcia-Yeguas

**Affiliations:** 1 Istituto Nazionale di Geofisica e Vulcanologia, Sezione di Pisa, Via Cesare Battisti, 53, 56125 Pisa, Italy; 2 School of Environmental Sciences, University of Liverpool, 4 Brownlow Street, Liverpool, L69 3GP UK; 3 University of Granada, Dpto. Didàctica de las Ciencias Experimentales, Facultad de Ciencias de la Educaciòn, Granada, Spain; 4 Instituto Andaluz de Geofìsica, Universidad de Granada, Granada, Spain

**Keywords:** Geophysics, Volcanology, Natural hazards

## Abstract

Infrasound is a valuable tool for volcano monitoring that can offer critical insights into volcanic processes, unrest and eruption. Over the last two decades, studies have shown the benefits of using local infrasound arrays - clusters of sensors near active vents - for real-time detection, tracking, and quantifying eruption intensity. This work introduces the first open-access dataset of infrasound array waveforms recorded at Mt. Etna, Italy, from May to October 2021 during intense eruptive activity. The 6-element array was installed at Monte Conca, near a permanent seismic station operated by the Istituto di Geofisica e Vulcanologia - Osservatorio Etneo, at about 6 km from the South-East Crater. The continuous monitoring captured 39 eruptions, successfully detecting all events, tracking their progression, and distinguishing activity from multiple vents. This manuscript details the array setup and summarizes volcanic activity during deployment, supporting future research and monitoring efforts. The dataset aims to aid development of early warning systems and serves as a valuable training tool for early-career scientists in the fields of volcano monitoring.

## Background *&* Summary

**Fig. 1:**
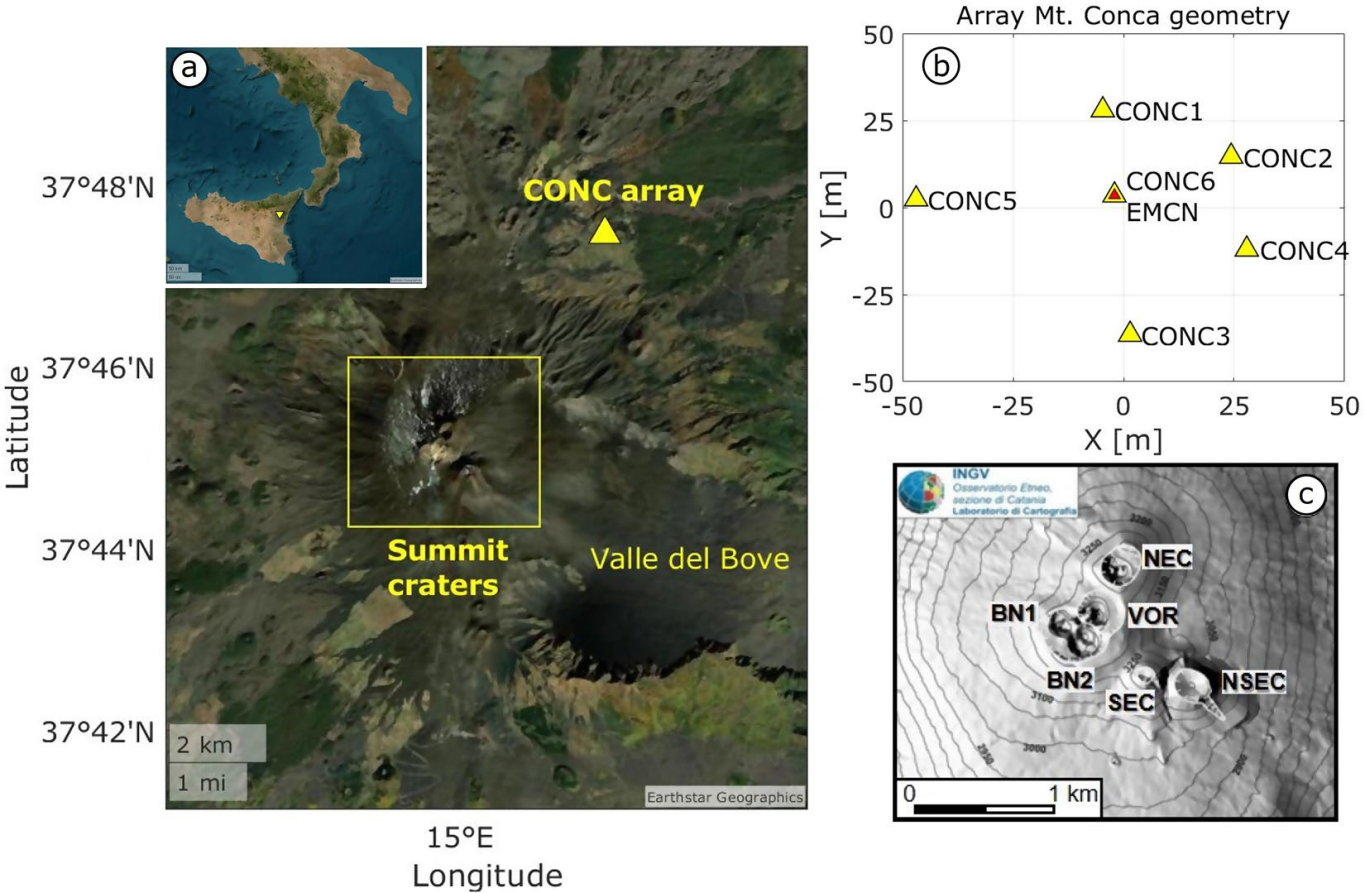
**a**) Map of Mt. Etna showing the location of the CONC infrasound array. The inset in the upper left corner indicates the position of Mt. Etna, Italy. (**b**) Configuration of the infrasound array; each yellow triangles mark the position of an individual infrasound sensor, while the red triangle represents the central node of the array, corresponding to the ECMP seismic station of the INGV network. (**c**) Close-up view of Mt. Etna’s summit craters.

Mt. Etna is one of the world’s most active volcanoes, characterized by frequent and intense eruptions over the past two decades, including extensive lava flows and significant ash emissions. The Istituto Nazionale di Geofisica e Vulcanologia - Osservatorio Etneo (INGV-OE) continuously monitors Etna with a real-time instrument network including seismic, acoustic, deformation, gas, and other remote sensing data. Mt. Etna’s consistent activity offers an ideal environment for assessing the performances of infrasound arrays for eruption detection and characterization, as well as for refining models that relate acoustic emissions to volcanic ash dispersal into the atmosphere (e.g., ref. ^1^). At Etna, volcanic infrasound - i.e., low-frequency acoustic waves typically in the 1-20 Hz range - is mostly generated by surface degassing and discrete explosions from the multiple summit vents. These signals can be recorded over a wide range of distances, from local (<15 km) to regional-scale (15–250 km)^2–4^. At local distances, infrasound sensors are configured as either arrays of microphones or distributed networks, with arrays offering greater noise suppression and source discrimination capabilities. Over the past two decades, the increased amount of infrasound data collected at active volcanoes has significantly enhanced our understanding of eruption dynamics, including their onset, progression, style, intensity, and associated hazards. Local^5^, but also global (>250 km)^4,6^ infrasound recordings have proven especially useful in developing real-time eruption detection methods. However, despite these advances, further progress is hindered by the limited availability of high-quality, and publicly accessible, datasets. Here, we present the first open-access infrasound array dataset collected during eruptive activity at Etna within the framework of a research collaboration between Istituto Nazionale di Geofisica e Vulcanologia - Sezione Pisa (INGV-PI), Istituto Nazionale di Geofisica e Vulcanologia - Osservatorio Etneo (INGV-OE) and the University of Liverpool (UK). A six-element infrasound array was installed at Monte Conca (CONC), near the permanent INGV-OE seismic station EMCN (Fig. 1), and operated continuously for 12 months, during a period of elevated eruptive activity. This manuscript introduces the infrasound dataset, including details on the instruments, deployment strategy, and data recovery statistics. We also present a summary of the most significant volcanic activity observed during the deployment period and provide examples of associated infrasound signals to facilitate future use of the dataset by other researchers. Finally, we show how infrasound array processing allows detection and discrimination of eruptive activity across the multiple summit vents at Etna. This dataset will provide valuable support to the development of fast and efficient real-time workflows for infrasound array processing and analysis, including uncertainty estimates for acoustic source locations, thereby promoting further integration of infrasonic array technology into volcano monitoring programs, and early warning protocols at Etna and other similar volcanoes worldwide.

### The 2021 eruptive activity at Mt. Etna

Etna typically exhibits persistent degassing from its summit craters and frequent eruptive activity from both summit and flank vents (e.g., refs. ^7,8^, and references therein). In recent years, eruptive activity at Etna has occurred from all its summit craters, sometimes simultaneously active. Five craters are located within the summit area (Fig. 1c): the Southeast Crater (SEC), the New Southeast Crater (NSEC), Bocca Nuova (BN), Voragine (VOR), and the Northeast Crater (NEC). A new phase of eruptive activity began in November 2020, marked by discrete Strombolian explosions accompanied by intermittent ash emissions and continuous degassing across the summit craters^8^. The activity escalated on 13 December 2020, when a sequence of more than 60 paroxysmal events - violent and sustained lava fountaining phenomena with ash column formation (e.g., ref. ^9^) - began continuing throughout 2021^10^. Each paroxysms typically began with discrete Strombolian activity - single burst-like volcanic eruptions with rhythmic and mild explosions (e.g., ref. ^11^) - evolving into sustained lava fountaining accompanied by ash emissions. Some eruptive plumes reached elevations of up to 10 km above the vent, with ash transported tens of kilometers downwind, impacting nearby towns and the Catania airport to the East. Paroxysms typically ended with a gradual waning of lava fountaining and transition to passive degassing, occasionally accompanied by lava effusion^12^. Our deployment period coincided with this eruptive activity; the infrasound array recorded 39 paroxysmal events from SEC/NSEC (see Table 1) as well as minor degassing and explosive activity from BN.

**Table 1:** Summary of Etna paroxysmal activity between May and October 2021 according to INGV Bollettini 2021 and ref. ^10^.

Number of Paroxysm	Date of the Paroxysmal event	Start activity (hh:mm) UTC	End of activity (hh:mm) UTC
20	19 May	01:15	05:15
21	21 May	01:05	02:55
22	22 May	20:40	22:40
23	24-25 May	20:25 (24/05)	03:00 (25/05)
24	25 May	18:20	19:35
25	26 May	01:55	03:50
26	26 May	10:35	11:30
27	27 May	no data	no data
28	28 May	06:47	07:25
29	28 May	15:40	16:15
30	28 May	20:07	20:50
31	30 May	04:40	05:45
32	02 June	08:30	10:45
33	04 June	16:05	17:30
34	12 June	13:50	23:15
35	14 June	21:35	22:45
36	16 June	11:50	12:50
37	17-18 June	20:20 (17/06)	00:10 (18/06)
38	19 June	18:30	20:20
39	20-21 June	22:15 (20/06)	00:15 (21/06)
40	22 June	02:55	04:15
41	23 June	02:15	03:20
42	23 June	18:00	19:00
43	24 June	09:55	10:45
44	25 June	01:00	01:50
45	25 June	18:25	19:15
46	26 June	15:50	17:00
47	27 June	08:25	09:48
48	28 June	15:00	15:30
49	01-02 July	22:50 (01/07)	00:50 (02/07)
50	04 July	15:25	17:00
51	06-07 July	22:30 (06-07)	00:20 (07/07)
52	08 July	20:45	22:50
53	14 July	10:40	12:30
54	20 July	05:50	08:48
55	31 July	19:15	23:30
56	08-09 August	23:10 (08/08)	04:00 (09/08)
57	29 August	15:45	20:40
58	21 September	07:55	09:30
59	23 October	08:45	10:20

Note that the numbering of paroxysms begins at 20, as those occurring prior to the installation date of our array have been excluded from the analysis.

## Methods

Over the last three decades, infrasound has been increasingly adopted to investigate eruption dynamics at active volcanoes, particularly at open-vent systems such as Mt. Etna (e.g., refs. ^1,6,13–19^). A permanent infrasound network of four stations was first installed at Etna in 2005, alongside the existing seismic network. Over the past 10 years, the number of stations has increased to 10, some co-located with permanent INGV seismic stations. However, the use of infrasound arrays and beamforming techniques has been more limited at Mt. Etna, with the exception of work by^5^. Typically, infrasound arrays have the ability to enhance signal detectability by increasing the signal-to-noise ratio (e.g., refs. ^1,14,20–24^).

The CONC (Monte Conca) infrasound array was the first of its kind deployed by INGV to monitor Mt. Etna’s activity. This deployment aimed primarily at conducting long-term testing of the technology before considering its integration into INGV-OE’s monitoring operations. A previous short-term array deployment (~2 months) was also conducted by the same team of researchers from the University of Liverpool (UK) and INGV^25^.

The five-element CONC array was installed on May 19, 2021, by a team of technicians and researchers from INGV-PI, INGV-OE, and the University of Liverpool (UK). On July 16, the final installation of a sixth sensor (CONC6) completed the planned six-element configuration, as shown in Fig. 1.

The array featured a central element co-located with the EMCN seismic station operated by INGV-OE, surrounded by five sensors with an aperture of approximately 70 meters (Fig. 1b). Table 2 details the coordinates of all elements within the CONC array. The array includes six IST2018 broadband infrasound sensors, with sensitivity of 20 mV/Pa, flat frequency response between 0.06 and 40 Hz, and a full-scale pressure range of +/−240 Pa; these sensors are developed by ISTerre (Universite Savoie Mont Blanc, France) and specifically designed for volcano monitoring. The sensors feature a compact, lightweight design (26 × 45 × 80 mm; 100 g) and low power consumption (42 mW), enabling easy deployment with minimal infrastructure. The IST2018 employs a cost-effective MEMS-based differential pressure transducer, offering reliable performance for field applications^26^. All data were recorded at 100 Hz using DIGOS DATA-CUBE^3^ digital recorders set to gain 1 (±4.096 V). At this gain the recorders offer an effective resolution of 22.4 bits, a dynamic range of 125 dB at 100 Hz, and GPS timing accuracy of <0.01 ms, ensuring high-precision during signal acquisition.

**Table 2:** Coordinates of the infrasonic sensors deployed at Monte Conca array site.

Array Monte Conca	Latitude (^°^)	Longitude (^°^)	Altitude (m)
CONC1	37.79131	15.03355	1869
CONC2	37.79119	15.03388	1864
CONC3	37.79073	15.03362	1883
CONC4	37.79095	15.03392	1875
CONC5	37.79108	15.03307	1876
CONC6	37.79109	15.03358	1865

**Fig. 2:**
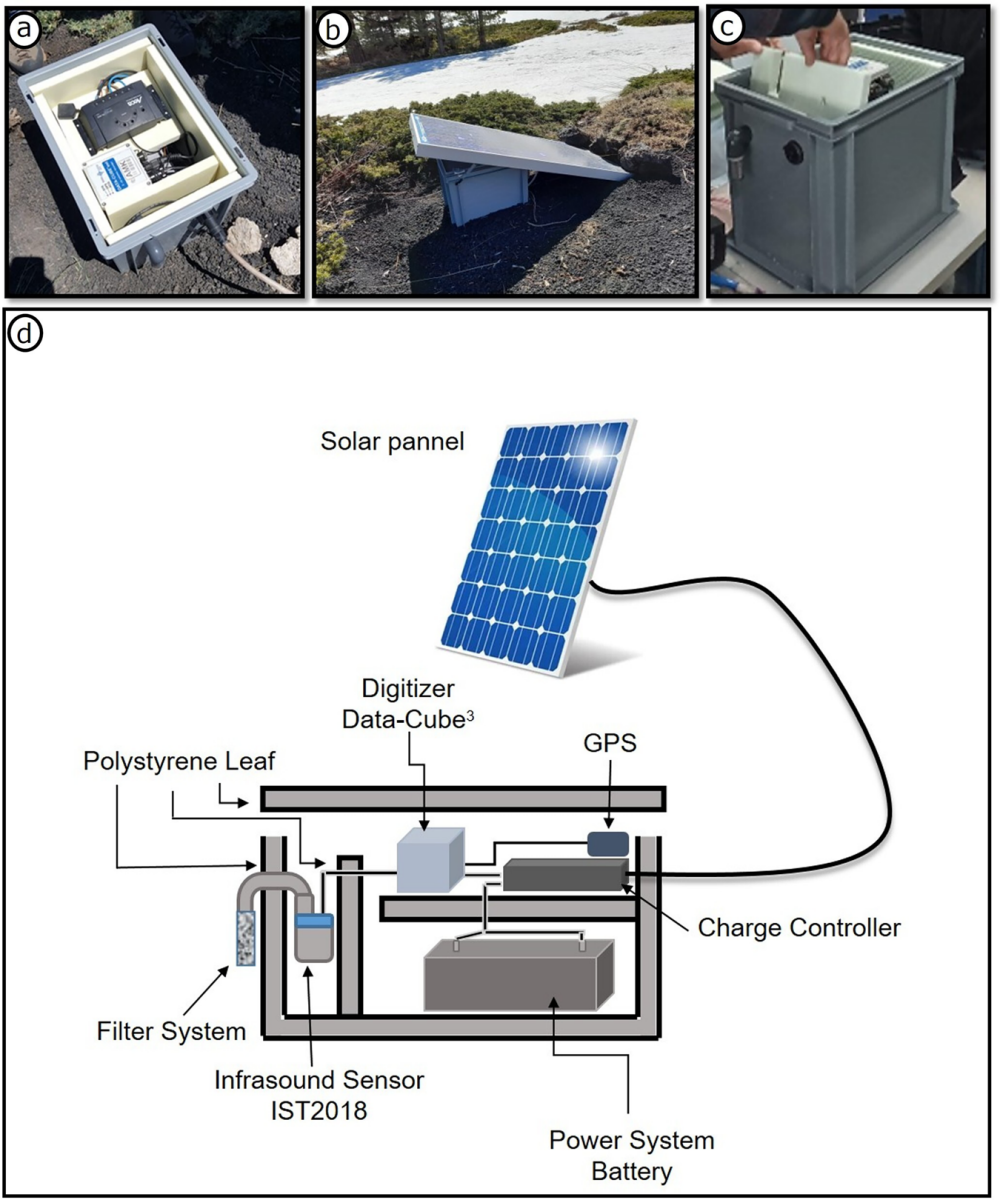
(**a**)–(**c**) Photos of the custom housing used for instrument deployment of the CONC array. (**d**) Sketch of the instrumental design.

The primary challenge of the deployment was ensuring continuous operation during the winter season, when Etna is covered by snow. A custom housing system was developed comprising a waterproof, thermally insulated, plastic box containing the sensor, digitizer, and a 12V/52Ah battery. An 80 W solar panel, mounted on the box and secured with steel springs to the ground, maintained sufficient battery voltage throughout the deployment. Each sensor was fitted with a mechanical wind noise filter protected by a stainless steel mesh guard; each box was lined with polyurethane foam (see Fig. 2). The design ensured thermal insulation, power stability, wind suppression and low visual impact, while supporting autonomous operation throughout the period of installation.

## Data Records

**Fig. 3:**
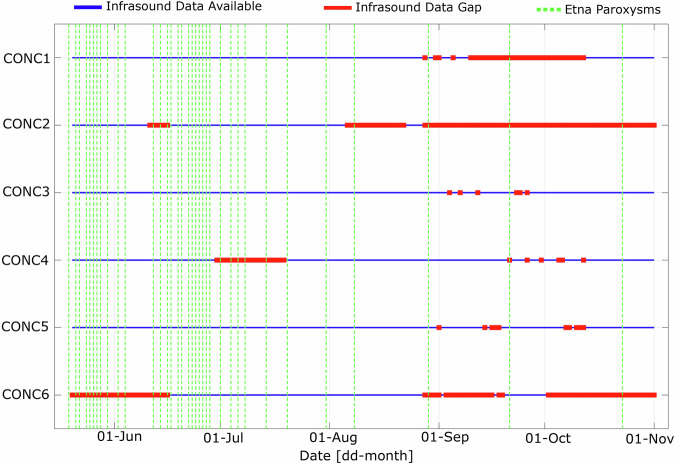
Data recovery statistics for the CONC infrasound array between May and November 2021, showing data availability for each of the six sensors. Blue lines indicate periods of available data; red segments mark data gaps. The extended gap for CONC2 reflects datalogger malfunctioning. Green-dashed lines indicate the time of paroxysmal activity at Mt. Etna.

The complete dataset, including raw waveforms and results of array processing, is available from FigShare repository ref. ^27^. Figure 3 illustrates data recovery statistics for the array during the period of deployment. The array operated continuously between May 19, 2021 and April 2, 2022, despite snow accumulation from mid-January to March. Snow did not significantly impact the power supply, allowing the system to mostly remain functional. Data recovery was generally high across the array, with the exception of sensor CONC2, which experienced data logger malfunctioning. Minor data gaps correspond to routine maintenance periods, including scheduled data downloads.

## Technical Validation

**Fig. 4:**
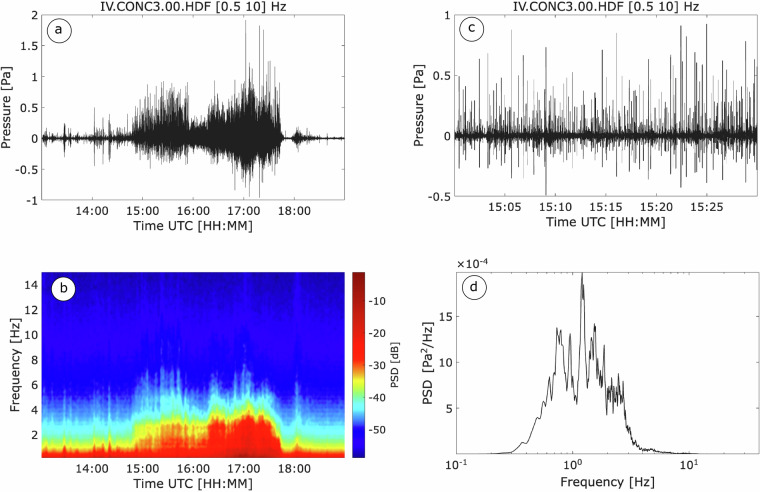
(**a**) Filtered infrasound waveform of the June 4 paroxysm at Mount Etna (Event No. 33, see Table 1), and (**b**) the corresponding spectrogram, showing energy concentration during the paroxysmal phase associated with Strombolian explosions and lava fountaining from the SEC/NSEC crater. (**c**) Sequence of Strombolian explosions during the paroxysm. (**d**) Spectra of all recorded explosions.

Figure 4a illustrates a representative filtered (0.5–10 Hz) waveform recorded at the CONC3 element of the array; these data illustrate six hours of infrasound on 4 June 2021 during paroxysmal event No. 33 (see Table 1).

The acoustic signal is characterized by continuous infrasonic tremor with varying properties, which can be classified into two main types: (i) tremor, likely associated to background continuous degassing activity, marked by generally low-amplitude, continuous waveforms not directly associated with eruptive phases (Fig. 4a,b, between 13:00 and 15:00 UTC); and (ii) pre-eruptive and syn-eruptive tremor, associated with paroxysmal activity (between 15:00 and 18:00 UTC). The latter typically consists of clusters of discrete explosion signals that progressively overlap until merging into a nearly continuous, high-amplitude waveform (see detail in Fig. 4c) during the most intense phase of lava fountaining.

It is possible to discriminate between the two phases of paroxysmal activity -Strombolian explosions and lava fountaining - based on their distinct spectral fingerprint. Discrete Strombolian explosions exhibit a clear spectral peak at approximately 1.5 Hz (Fig. 4d), while the lava fountain infrasound observed at 16:30 UTC displays a broader frequency content (see Fig. 4b).

**Fig. 5:**
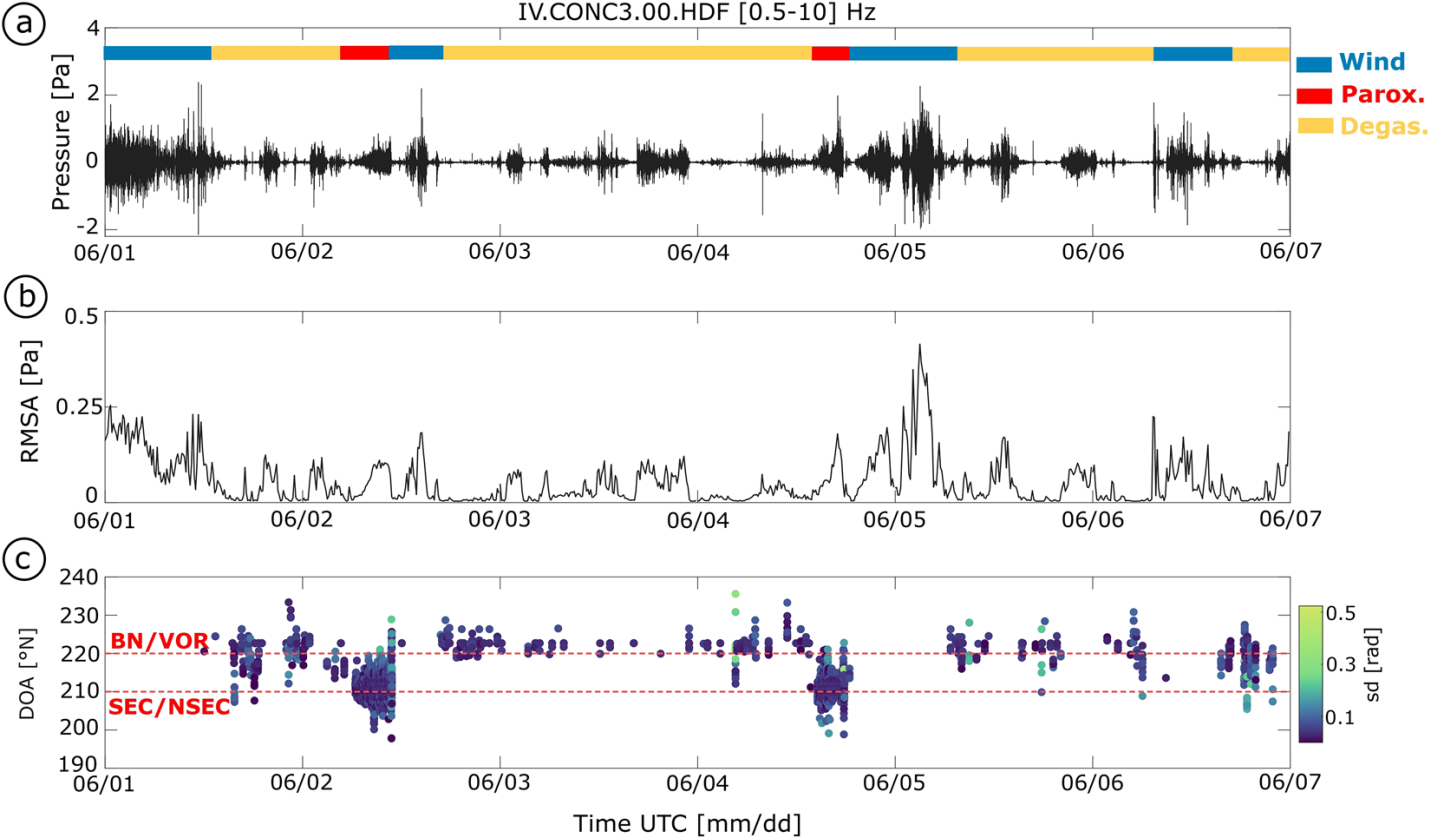
Example of infrasonic data recorded by the CONC array between June 1 and 6, 2021, encompassing paroxysms No. 32 and 33 (see Table 1). (**a**) Bandpass-filtered waveforms (0.5-10 Hz frequency band) showing both low-amplitude, non-eruptive tremor and high-amplitude, paroxysmal signals. (**b**) Root Mean Square Amplitude (RMSA), computed over 10-minute windows with 90% overlap. (**c**) DOA results with two horizontal dashed lines indicating the mean direction of arrivals (DOA) for SEC/NSEC (lower line) and BN/VOR (upper line). Only detections with high coherence (>80%) are shown, color-coded by standard deviation of the DOA estimate.

Figure 5 shows the value of array processing in isolating coherent volcanic infrasound and distinguishing activity at different summit craters during the first week of June 2021, which includes paroxysmal event No. 32 (see Table 1). Based solely on the filtered infrasound waveform and RMSA recorded continuously by the CONC array (Fig. 5a,b), the identification of signals associated with volcanic activity is challenging. The RMSA, computed over 10-minute windows with 90% overlap, exhibits peaks not only during eruptive phases but also during periods of elevated noise, particularly wind, making its interpretation ambiguous. Array processing allows detection and characterization of coherent infrasound signals across all array sensors, suppressing incoherent background noise such as from wind^28^. At Mt. Etna, where noise levels fluctuate significantly, arrays equipped with wind noise reduction systems (WNRs) can be critical for reliable monitoring (see Fig. 2). Our analysis adopts beamforming methods routinely used in infrasound studies (e.g., refs. ^29–31^), using time-domain, multi-channel cross-correlation over sliding time windows. In this study, we used 10-second processing windows with 90% overlap (i.e., a 1-second shift), and filtered the data between 1 and 10 Hz. Within each window, we computed cross-correlations between sensor pairs and quantified signal coherence based on peak correlation values. When a coherent signal was detected, we extracted key wave parameters: pressure amplitude, direction of arrivals (DOA), and apparent velocity. The pressure amplitude is defined as the peak value within the window, while the DOA and apparent velocity are derived from time delays between sensors^32^. The DOA, expressed as the azimuthal angle clockwise from true North, represents the horizontal direction of wave propagation.

Figure 5c shows DOA estimates for detections with high coherence (>80%), color-coded by their standard deviation. The standard deviation of the DOA estimates was calculated using the circular standard deviation (*circ_std* in MATLAB), which accounts for the periodic nature of angular data. These results reveal consistent clusters of coherent detections from spatially distinct source regions. Two dominant DOA clusters emerge, corresponding to infrasound emissions from the SEC-NSEC during paroxysmal phases and from BN/VOR during periods of degassing. During the paroxysmal event, DOA estimates consistently point toward the SEC/NSEC, confirming its role as the dominant source. Outside these events, detections shift toward stable azimuths associated with BN/VOR, which were active during that period. These stable directional patterns demonstrate the array’s ability to resolve overlapping volcanic signals over time. Notably, during high-noise periods, no detections are observed, as the signals are incoherent and therefore not attributable to volcanic activity.

**Fig. 6:**
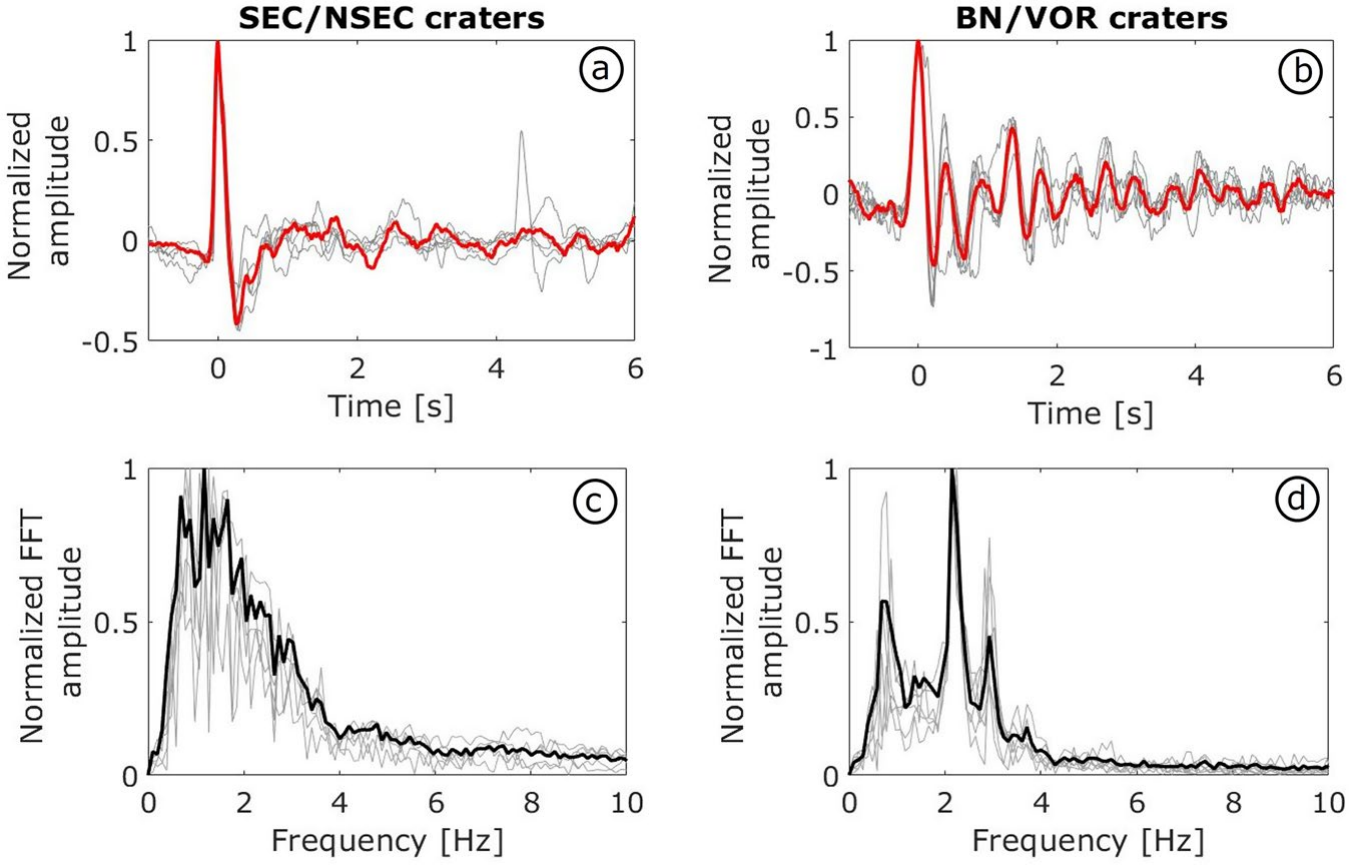
Waveform and spectral characteristics of infrasonic signals from South-East crater and Bocca Nuova/ Voragine craters. (**a**–**c**). Representative normalized infrasonic waveforms from seven explosive events at the SE and BN/VOR craters are shown. Individual waveforms are depicted in light grey, while the ensemble average is indicated in red. (**b**–**d**) Mean power spectral density (PSD) of the SE/NSEC and BN/VOR crater events (black lines), averaged over all waveforms (light grey lines).

By exploiting the detections, we are able to discriminate and extract coherent signals associated with explosive activity from different volcanic sources during the analyzed period. Figure 6 presents examples of infrasound waveforms and their spectral content from both the SEC/NSEC and the BN/VOR active craters. The SEC/NSEC waveforms (Fig. 6a) are characterized by an impulsive onset followed by a rapid amplitude decay, typical of explosive bursts of pressurized gas^33^. In contrast, the BN/VOR waveforms (Fig. 6b) exhibit sharp pulse followed by more oscillatory and longer-lasting codas. The SEC/NSEC signals display braod spectral energy concentrated between 1 and 2 Hz, whereas the BN/VOR events show distinct spectral peaks near 1, 2, and 3 Hz, indicative of acoustic resonance phenomena influenced by vent and crater morphology^34^.

Our experiment demonstrates the feasibility and robustness of infrasound array deployments at Mt. Etna for monitoring volcanic activity. The successful installation and operation of a six-element array at approximately 1800 m above sea level (a.s.l.) enabled the continuous acquisition of high-quality infrasound data over a year-long period, including paroxysmal activity phases. The data collected provide a unique record of both eruptive and passive degassing activity, offering new opportunities to investigate infrasound signal characteristics and their relation to different eruptive processes. Furthermore, the experiment served as a valuable testing ground for infrasound array processing workflows, confirming their potential to support real-time monitoring and source localization. This work represents a critical step toward enhancing acoustic surveillance capabilities at Etna and at volcanoes worldwide, and improving eruption early warning systems.

## Data Availability

The full dataset, including continuous raw waveform data and array information, is openly available in the FigShare ref. ^27^.

## Code availability

The code used to visualize all raw waveforms in the dataset is likewise openly available in the FigShare ref. ^27^.

## References

[1] De Angelis, S., Zuccarello, L., Scollo, S. & Mereu, L. Assessment of eruption source parameters using infrasound and plume modelling: a case study from the 2021 eruption of Mt. Etna, Italy. *Sci. Rep.* **13**, 19857, 10.1038/s41598-023-46160-6 (2023). 37963914 10.1038/s41598-023-46160-6 PMC10645732

[2] Marchetti, E. *et al*. Long range infrasound monitoring of Etna volcano. *Sci. Rep.* **9**, 1–10, 10.1038/s41598-019-54468-5 (2019). 31784608 10.1038/s41598-019-54468-5 PMC6884589

[3] Gheri, D. *et al*. Monitoring of Indonesian volcanoes with the IS06 infrasound array. *J. Volcanol. Geotherm. Res*. 107753, 10.1016/j.jvolgeores.2023.107753 (2023)

[4] Fee, D. & Matoza, R. S. An overview of volcano infrasound: From Hawaiian to Plinian, local to global. *J. Volcanol. Geotherm. Res.* **249**, 123–139, 10.1016/j.jvolgeores.2012.09.002 (2013).

[5] Ripepe, M. *et al*. Infrasonic early warning system for explosive eruptions. *J. Geophys. Res. Solid Earth* **123**, 9570–9585, 10.1029/2018JB015561 (2018).

[6] Gheri, D. *et al*. Detecting explosive volcanism using global long-range infrasound data. *J. Volcanol. Geotherm. Res.* **462**, 108320, 10.1016/j.jvolgeores.2025.108320 (2025).

[7] Corsaro, R. A. *et al*. Monitoring the December 2015 summit eruptions of Mt. Etna. *J. Volcanol. Geotherm. Res.* **341**, 53–69, 10.1016/j.jvolgeores.2017.04.018 (2017).

[8] Andronico, D., Cannata, A., Di Grazia, G. & Ferrari, F. The 1986–2021 paroxysmal episodes at the summit craters of Mt. Etna: Insights into volcano dynamics and hazard. *Earth-Sci. Rev.* **220**, 103686, 10.1016/j.earscirev.2021.103686 (2021).

[9] Parfitt, E., Wilson, L. & Kerber, L.*Fundamentals of Physical Volcanology*. (John Wiley & Sons, 2025).

[10] Proietti, C., De Beni, E., Cantarero, M., Ricci, T. & Ganci, G. Rapid provision of maps and volcanological parameters: Quantification of the 2021 Etna volcano lava flows through the integration of multiple remote sensing techniques. *Bull. Volcanol.* **85**(10), 58, 10.1007/s00445-023-01673-w (2023).

[11] Taddeucci, J., Edmonds, M., Houghton, B., James, M. R. & Vergniolle, S. Hawaiian and Strombolian eruptions. In *The Encyclopedia of Volcanoes* (eds. Sigurdsson, H. *et al*.) 485-503, 10.1016/B978-0-12-385938-9.00027-4 (Elsevier, 2015).

[12] Calvari, S., Bonaccorso, A. & Ganci, G. Anatomy of a paroxysmal lava fountain at Etna volcano: The case of the 12 March 2021 episode. *Remote Sens.* **13**(15), 3052, 10.3390/rs13153052 (2021).

[13] Braun, T. & Ripepe, M. Interaction of seismic and air waves recorded at Stromboli volcano. *Geophys. Res. Lett.* **20**(1), 65–68, 10.1029/92GL02543 (1993).

[14] Ripepe, M. & Marchetti, E. Array tracking of infrasonic sources at Stromboli volcano. *Geophys. Res. Lett.* **29**(22), 33–1, 10.1029/2002GL015452 (2002).

[15] Fee, D., Garces, M. & Steffke, A. Infrasound from Tungurahua volcano 2006-2008: Strombolian to Plinian eruptive activity. *J. Volcanol. Geotherm. Res.* **193**(1-2), 67–81, 10.1016/j.jvolgeores.2010.03.006 (2010).

[16] Fee, D. *et al*. Infrasonic harmonic tremor and degassing bursts from Halema uma u crater, Kīlauea volcano, Hawaii. *J. Geophys. Res. Solid Earth* **115**B11, 10.1029/2010JB007642 (2010).

[17] Barfucci, G. & Ripepe, M. Dome collapse interaction with the atmosphere. *Geophys. Res. Lett.* **45**(17), 8923–8930, 10.1029/2018GL078243 (2018).

[18] De Angelis, S., Diaz-Moreno, A. & Zuccarello, L. Recent developments and applications of acoustic infrasound to monitor volcanic emissions. *Remote Sens.* **11**(11), 1302, 10.3390/rs11111302 (2019).

[19] Sciotto, M. *et al*. Infrasonic gliding reflects a rising magma column at Mount Etna (Italy). *Sci. Rep.* **12**, 16954, 10.1038/s41598-022-20258-9 (2022). 36261590 10.1038/s41598-022-20258-9 PMC9582027

[20] Ripepe, M., Marchetti, E. & Ulivieri, G. Infrasonic monitoring at Stromboli volcano during the 2003 effusive eruption: Insights on the explosive and degassing process of an open conduit system. *J. Geophys. Res. Solid Earth* **112**, B9, 10.1029/2006JB004613 (2007).

[21] Cannata, A., Montalto, P., Privitera, E. & Russo, G. Characterization and location of infrasonic sources in active volcanoes: Mount Etna, September-November 2007. *J. Geophys. Res. Solid Earth* **114**, B8, 10.1029/2008JB006007 (2009).

[22] Cannata, A. *et al*. Long period and very long period events at Mt. Etna volcano: Characteristics, variability and causality, and implications for their sources. *J. Volcanol. Geotherm. Res.* **187**(3-4), 227–249, 10.1016/j.jvolgeores.2009.09.007 (2009).

[23] Marchetti, E., Ripepe, M., Ulivieri, G., Caffo, S. & Privitera, E. Infrasonic evidences for branched conduit dynamics at Mt. Etna volcano, Italy. *Geophys. Res. Lett*. **36**, 19, 10.1029/2009GL040070 (2009).

[24] Montalto, P., Nunnari, G., Cannata, A., Privitera, E. & Partanè, D. Clustering of infrasonic events as tool to detect and locate explosive activity at Mt. Etna volcano. In From Physics To Control Through An Emergent View 195–200, 10.1142/9789814313155_0029 (World Scientific, 2010).

[25] De Angelis, S., Zuccarello, L., Rapisarda, S. & Minio, V. Introduction to a community dataset from an infrasound array experiment at Mt. Etna, Italy. *Sci. Data* **8**, 247, 10.1038/s41597-021-01030-6 (2021). 34556660 10.1038/s41597-021-01030-6 PMC8460648

[26] Grangeon, J. & Lesage, P. A robust, low-cost and well-calibrated infrasound sensor for volcano monitoring. *J. Volcanol. Geotherm. Res.* **387**, 106668, 10.1016/j.jvolgeores.2019.106668 (2019).

[27] Gheri, D., Zuccarello, L., De Angelis, S. & García-Yeguas, A. Infrasound recordings from 2021 eruptive episodes of Etna volcano 10.6084/m9.figshare.29037734 (2025).

[28] Christie, D. & Campus, P. The IMS infrasound network: Design and establishment of infrasound stations. In *Infrasound Monitoring for Atmospheric Studies* 29–75 (Springer, 2010).

[29] Cansi, Y. An automatic seismic event processing for detection and location: The PMCC method. *Geophys. Res. Lett.* **22**(9), 1021–1024, 10.1029/95GL00468 (1995).

[30] Hupe, P., Ceranna, L., Le Pichon, A., Matoza, R. S. & Mialle, P. International monitoring system infrasound data products for atmospheric studies and civilian applications. *Earth Syst. Sci. Data* **14**(9), 4201–4230, 10.5194/essd-14-4201-2022 (2022).

[31] Poste, B. *et al*. The multichannel maximum-likelihood (MCML) method: A new approach for infrasound detection and wave parameter estimation. *Geophys. J. Int.* **232**(2), 1099–1112, 10.1093/gji/ggac377 (2023).

[32] Bishop, J. W., Fee, D. & Szuberla, C. A. Improved infrasound array processing with robust estimators. *Geophys. J. Int.* **221**(3), 2058–2074, 10.1093/gji/ggaa110 (2020).

[33] Matoza, R. S., Fee, D. & López, T. M. Acoustic characterization of explosion complexity at Sakurajima, Karymsky, and Tungurahua volcanoes. *Seismol. Res. Lett.* **85**(6), 1187–1199, 10.1785/0220140110 (2014).

[34] Johnson, J. B., Watson, L. M., Palma, J. L., Dunham, E. M. & Anderson, J. F. Forecasting the eruption of an open-vent volcano using resonant infrasound tones. *Geophys. Res. Lett.* **45**(5), 2213–2220, 10.1002/2017GL076506 (2018).

